# Cardiofaciocutaneous syndrome and immunodeficiency: data from an international multicenter cohort

**DOI:** 10.3389/fimmu.2025.1598896

**Published:** 2025-07-07

**Authors:** Benedetta Elena Di Majo, Chiara Leoni, Eleonora Cartisano, Chiara Fossati, Germana Viscogliosi, Valentina Trevisan, Lucia Pia Bruno, Francesca Conti, Mattia Moratti, Emilia Monaco, Donato Rigante, Beatrice Rivalta, Caterina Cancrini, Aleksandra Szczawińska-Popłonyk, Aleksander Jamsheer, Monika Obara-Moszyńska, Viktoria Zakharova, Anna Shcherbina, Julija Rodina, Beyhan Tüysüz, Saumya Shekhar Jamuar, Jiin Ying Lim, Jeannette Goh, Anna Cereda, Teresa Agovino, Ilaria Contaldo, Maria Luigia Gambardella, Adriana Cristina Balduzzi, Alessia Cherubino, Giovanni Antonio Marrocco, Silvia Bellesi, Valentina Carusi, Gabriele Rumi, Andrea Biondi, Giuseppe Zampino, Francesco Saettini

**Affiliations:** ^1^ Pediatria, Fondazione IRCCS San Gerardo dei Tintori, Monza, Italy; ^2^ Dipartimento Di Medicina e Chirurgia, Università Degli Studi Milano-Bicocca, Monza, Italy; ^3^ Center for Rare Diseases and Birth Defects, Department of Woman and Child Health and Public Health, Fondazione Policlinico A. Gemelli, IRCCS, Rome, Italy; ^4^ Pediatric Unit, IRCCS Azienda Ospedaliero-Universitaria di Bologna, Bologna, Italy; ^5^ Department of Life Sciences and Public Health, Fondazione Policlinico Universitario A. Gemelli IRCCS, Rome, Italy; ^6^ Università Cattolica Sacro Cuore, Rome, Italy; ^7^ Research and Clinical Unit of Primary Immunodeficiencies, IRCCS Bambino Gesù Children’s Hospital, Rome, Italy; ^8^ Clinical Unit of Clinical Immunology and Vaccinology, IRCCS Bambino Gesù Children’s Hospital, Rome, Italy; ^9^ Chair of Pediatrics, Department of Systems Medicine, University of Rome “Tor Vergata”, Roma, Italy; ^10^ Department of Pediatric Pneumonology, Allergy and Clinical Immunology, Institute of Pediatrics, Poznań University of Medical Sciences, Poznań, Poland; ^11^ Department of Medical Genetics, Poznań University of Medical Sciences, Poznań, Poland; ^12^ Diagnostyka GENESIS, Poznań, Poland; ^13^ Department of Pediatric Endocrinology and Rheumatology, Institute of Pediatrics, Poznan University of Medical Sciences, Poznan, Poland; ^14^ Clinical Data Analysis Department, National Medical Research Center for Endocrinology, Moscow, Russia; ^15^ Department of Immunology, Dmitry Rogachev National Medical Research Center of Pediatric Hematology, Oncology and Immunology, Moscow, Russia; ^16^ Department of Pediatric Genetics, Cerrahpasa Medical School, Istanbul University-Cerrahpasa, Istanbul, Türkiye; ^17^ Genetics Service, KK Women’s and Children’s Hospital, Singapore, Singapore; ^18^ International Rare Disease Research Consortium, Paris, France; ^19^ Department of Pediatric, “Papa Giovanni XXIII” Hospital, Bergamo, Italy; ^20^ Child Neurology and Psychiatric Unit, Department of Woman and Child Health and Public Health, Fondazione Policlinico Universitario A. Gemelli, IRCCS, Rome, Italy; ^21^ Dipartimento di Scienze di Laboratorio ed Ematologiche, Fondazione Policlinico Gemelli, IRCCS, Rome, Italy; ^22^ UOSD Allergologia ed Immunologia Clinica, Dipartimento Scienze Mediche e Chirurgiche, Fondazione Policlinico Universitario A. Gemelli, IRCCS, Rome, Italy; ^23^ Unità Operativa Semplice Malattie Infiammatorie Croniche Intestinali, Inflammatory Bowel Disease (IBD) Unit, CEMAD, Fondazione Policlinico Universitario A. Gemelli, IRCCS, Rome, Italy; ^24^ Centro Tettamanti, Fondazione IRCCS San Gerardo dei Tintori, Monza, Italy

**Keywords:** primary immunodeficiency, inborn errors of immunity, cardiofaciocutaneous syndrome, rasopathy, hypogammaglobulinemia, BRAF, MAP2K1, Syndromic immunodeficiency

## Abstract

**Introduction:**

Cardiofaciocutaneous syndrome (CFCS) is a rare syndromic disorder caused by germline mutations affecting the RAS/MAPK pathway. It is characterized by distinctive craniofacial dysmorphism, congenital heart defects, skin abnormalities, gastrointestinal dysfunction, neurocognitive impairment, and epilepsy. Emerging evidence suggests an association with hypogammaglobulinemia, but a comprehensive characterization of immunological abnormalities in CFCS is lacking.

**Methods:**

We conducted a retrospective, multicenter observational study to investigate the immunological phenotype of CFCS. Clinical features, immune-related manifestations, and laboratory parameters were analyzed to delineate the immunological profile of affected individuals.

**Results:**

A total of 56 patients with a confirmed clinical and molecular diagnosis of CFCS were included, with a median age at evaluation of 13 years (range: 1–39 years). Increased susceptibility to infections was reported in 18/56 patients (32%), while autoimmune manifestations were observed in 14/56 patients (25%). Common immunological findings included monocytosis (32%), lymphopenia (21%), and hypogammaglobulinemia, with decreased IgG, IgA, or IgM levels in 21%, 40%, and 35% of patients, respectively. Genotype-phenotype analysis revealed that *BRAF* mutations were predominantly associated with T-cell lymphopenia, whereas *MAP2K1* mutations were linked to monocytosis, reduced naïve and switched-memory B cells, and hypogammaglobulinemia. Immunodeficiency-related treatments, including immunoglobulin replacement therapy, antibiotic prophylaxis, or immunosuppressive therapy, were administered to 6/56 patients (11%).

**Conclusions:**

CFCS is associated with recurrent yet heterogeneous immunological abnormalities, including lymphopenia, hypogammaglobulinemia, and increased infection susceptibility. Given these findings, routine immunological assessment should be considered in CFCS patients to facilitate early detection and appropriate management of immune dysfunction.

## 1 Introduction

Germline mutations in genes encoding proteins of the RAS/MAPK signaling pathway give rise to a family of clinically related disorders, collectively known as RASopathies. Among these, cardiofaciocutaneous syndrome (CFCS; OMIM #115150, #615278, #615279 and #615280) is a rare autosomal dominant disease that affects 1/800,000 newborns (1) and it is caused by mutations in *BRAF*, *MAP2K1*, *MAP2K2*, *KRAS* and *YWHAZ* genes (2). CFCS is characterized by a distinctive combination of dysmorphic craniofacial features, heart and skin abnormalities, gastrointestinal dysfunction, neurocognitive delay and epilepsy (3, 4).

Inborn errors of immunity (IEI) are heterogeneous conditions that share increased susceptibility to infections, autoimmunity, allergy, autoinflammation and predisposition to malignancy (5, 6). Syndromic immunodeficiencies are a subgroup of IEI characterized by involvement of different organ systems simultaneously (7).

Recently, case reports have described the immunological phenotype of CFCS, reporting antibody deficiency in patients affected by *MAP2K1* and *MAP2K2* mutations (8–10). To date, there are no studies that precisely portray the immunological phenotype of CFCS, its natural history, and treatment for immunological defects.

Here we report the results of a multicenter retrospective observational study on the immunological characterization of patients with clinical and molecular confirmed diagnosis of CFCS. We aim to investigate clinical and immunological features, including rare, severe or novel phenotypes, gather data on employed treatments, and identify correlations with non-immunological traits.

## 2 Methods

One author (C.E.) carried out a systematic review of all studies published as original articles up to May 31, 2024, in PubMed database. “Cardiofaciocutaneous syndrome”. Languages included were English, Spanish, and French. The corresponding authors of all relevant publications and referring physicians were contacted to obtain updated clinical information on the reported cases. To be included in our study, the criteria were: clinical diagnosis of CFCS, pathogenic or variants of unknown significance in CFCS genes (*BRAF*, *MAP2K1*, *MAP2K2*, *KRAS*, *YWHAZ*), completion of the Case Report Form (CRF) and provided written informed consent either from the participant, parents or legal guardians.

Data was obtained from the available medical records and collected using a CRF. Collected data included: demographics, genetic diagnosis, dysmorphisms and organ malformation, other features of CFCS (e.g., intellectual disability, epilepsy), immunological manifestations (increased susceptibility to infections [either severe (11) or recurrent (12)], autoimmunity, lymphoproliferation), immunological tests including complete blood count (CBC) with differential, immunoglobulin levels (IgG, IgA, IgM), lymphocyte subsets, serological response to vaccinations, serum autoantibodies, treatment and need for hospitalization. Lymphocyte subsets distribution and immunoglobulin levels were classified as normal, high or low according to published age-matched reference values (13–15).

The study was approved by the local hospital Ethical Committee (PID-GENMET) and was conducted according to the Helsinki Declaration.

### 2.1 Statistics

Possible associations between the baseline clinical features and the finding of each one of the laboratory or clinical findings were assessed via Pearson’ chi-square test or Fisher exact test. Correlation among variables included in the study was analyzed through the measurement of Pearson’s correlation coefficient r (ranging from − 1 to 1). The strength of the correlation between the two variables was considered “none” when r value was < 0.3, “weak” when 0.3 ≥ r value < 0.5, “moderate” when 0.5 ≥ r value < 0.7 and “strong” when r value ≥ 0.7. Results were deemed significant for p value < 0.05 with a double-tailed test.

## 3 Results

### 3.1 Systematic review

The PubMed search for papers describing the CFCS produced 247 results. Ninety-one papers were excluded as irrelevant (i.e. languages other than the selected ones = 1, papers not describing patients, papers describing different conditions; **Supplementary Figure 1**). Of the remaining 156 papers, all the 130 corresponding authors were contacted and eight of them provided responses and data. Referring physicians provided updated information (when available) and data on unpublished patients.

### 3.2 Patients’ characteristics

**Figure 1:**
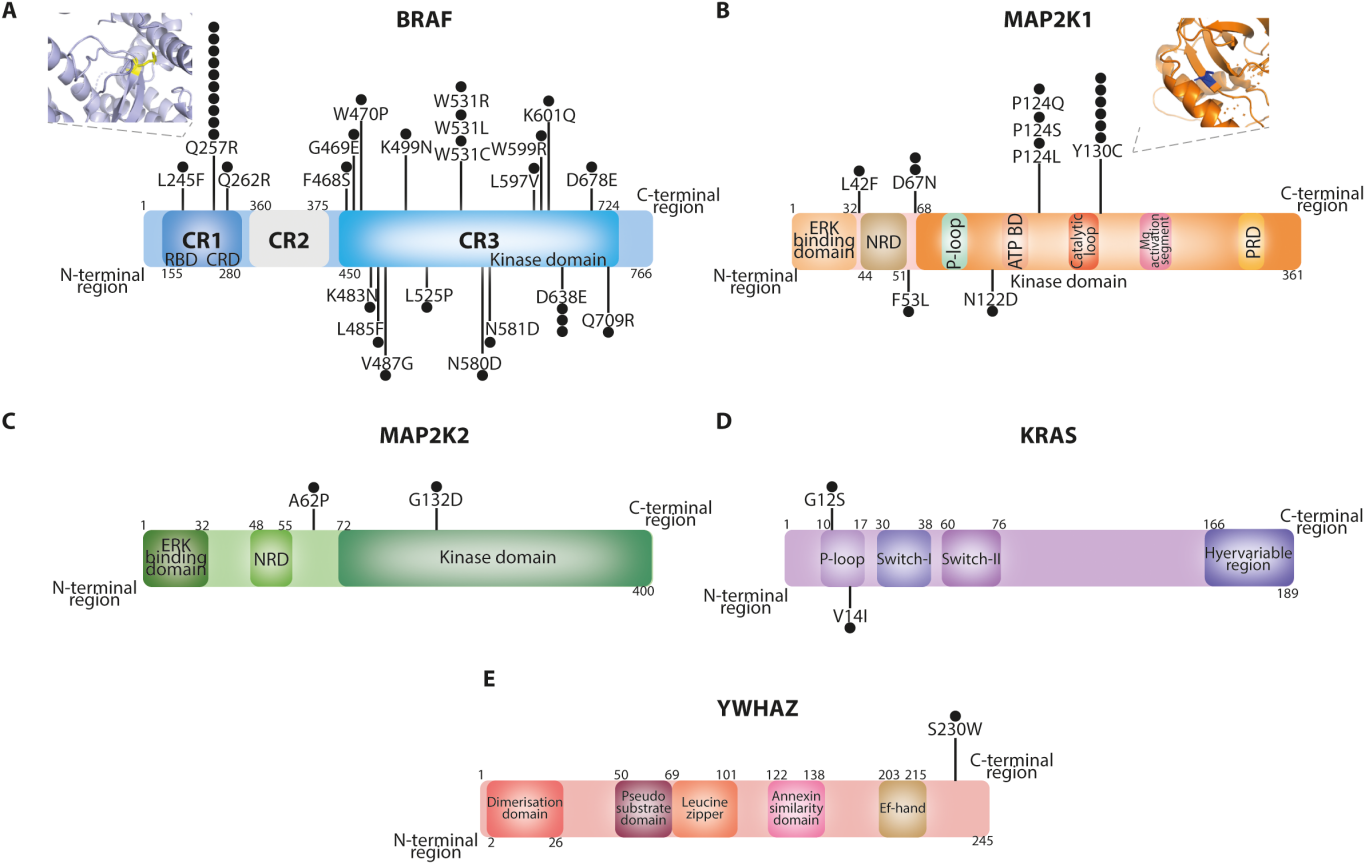
Protein domains of *BRAF* **(A)**, *MAP2K1* **(B)**, *MAP2K2* **(C)**, *KRAS* **(D)** and *YWHAZ* **(E)** proteins highlighting the mutations identified in our cohort. The *BRAF* Q257R and *MAP2K1* Y130C variants were modelled through PyMOL based on the PDB structures 7mfe and 3w8q, respectively. Each black dot indicates an affected patient.

A total of 56 patients with molecularly confirmed CFCS were included in the final cohort for analysis. Of the 56 patients included, 51 carried pathogenic variants, 4 had likely pathogenic variants, and 1 had a variant of uncertain significance (VUS). In cases with likely pathogenic variants or a VUS, the diagnosis of CFCS was confirmed by an expert clinical geneticist based on the overall phenotype and current variant interpretation guidelines. Demographic data and CFCS-associated anomalies are summarized in **Table 1**. The median age at the last follow-up was 13 years (range 1–39 years), with a predominance of female patients (females, 62.5%). Molecular diagnosis was primarily achieved through next-generation sequencing (83.6%), while whole exome sequencing (9.1%), Sanger sequencing (5.5%), and whole genome sequencing (1.8%) were used less frequently. In one case, the specific molecular technique could not be retrieved from the patient’s medical records. Among the cohort, 37 patients (66.1%) harbored variants in *BRAF*, 14 (25.0%) in *MAP2K1*, 2 (3.6%) in *MAP2K2*, 2 (3.6%) in *KRAS*, and 1 (1.8%) in *YWHAZ* (**Figure 1**). All the variants were missense mutations detected in heterozygous state. In one patient the variant p.(Phe35Leu) in *MAP2K1* was found in a mosaic state (9). In the entire cohort, genetic testing identified BRAF mutations in 37 patients, including 34 with pathogenic variants and 3 with likely pathogenic variants. MAP2K1 mutations were found in 14 patients, all classified as pathogenic. One patient carried a pathogenic variant in MAP2K2, while another patient harbored a variant of uncertain significance (VUS) in the same gene. KRAS mutations were identified in two individuals, both pathogenic. Finally, one patient had a VUS in YWHAZ. Overall, 51/56 variants (91.1%) were classified as pathogenic, 4/56 (7.1%) as likely pathogenic, and 1/56 (1.8%) as a VUS.

**Table 1:** Demographic characteristics, genetic data and CFCS associated anomalies in the whole cohort.

Age range (median)	1–39 years	13 years
	Patients (Total)	%
Males	21 (56)	37.5
Alive	56	100
Diagnosis		
Whole genome sequencing	1 (55)	1.8
Whole exome sequencing	5 (55)	9.1
Next generation sequencing	46 (55)	83.6
Sanger sequencing	3 (55)	5.5
Genetic data		
*BRAF*	37 (56)	66.1
*MAP2K1*	14 (56)	25.0
*MAP2K2*	2 (56)	3.6
*KRAS*	2 (56)	3.6
*YWHAZ*	1 (56)	1.8
CFCS-associated phenotype		
**Skin**	36 (53)	67.9
Dermatitis	9	17.0
Other skin manifestations	26	49.1
**CNS**	51 (54)	94.4
Psychomotor or intellectual Delay, ASD	47	87.0
Epilepsy	25	46.3
Other	16	29.6
**Heart**	44 (55)	80.0
Valve	19	34.6
Pulmonary stenosis	15	27.3
Hypertrophy	9	16.4
Other	13	23.6
**Gastrointestinal**	26 (45)	57.8
Feeding difficulties	19	42.2
GERD and vomiting	10	22.2
Other	8	17.8
**Ocular**	35 (49)	71.4
Nystagmus	3	6.1
Extrinsic muscular defects	27	55.1
Refractive defects	18	36.7
Other	6	12.2
**Endocrine**	23 (52)	44.2
GH deficiency	7	13.5
Growth delay	10	19.2
Other	8	15.4
**Musculoskeletal**	36 (50)	72

ASD, autism Spectrum Disorder; CNS, central nervous system; GERD, gastroesophageal reflux disease; GH, growth hormone.

Bold values indicate the major organ systems assessed for CFCS-associated clinical manifestations.

### 3.3 Infections

Data regarding severe or recurrent infections was available for all patients and is summarized in **Table 2**; **Supplementary Table 1**. Eighteen out of 56 patients (32.1%) had a history of severe or recurrent infections, predominantly affecting the upper and lower respiratory tracts (50%) or the gastrointestinal tract (8.9%). Hospitalization for infection management was required in ten individuals (17.9%), primarily due to sepsis, pneumonia or gastroenteritis. Additionally, eleven patients (19.6%) had a history of recurrent infections, including respiratory tract infections (otitis, URTI and LRTI), urinary tract infections and gastroenteritis.

**Table 2:** Immunological clinical characteristics and treatment strategy in the whole cohort.

	Patients (Total)	%
Infections	18 (56)	32.1
Severe	10	17.9
Recurrent	11	19.6
Autoimmune manifestations	14 (56)	25.0
Lymphoproliferation	0 (56)	0.0
Malignancies	0 (56)	0.0
Treatment for immunodeficiency	6 (56)	10.7

### 3.4 Autoimmune or immune dysregulation manifestations

Features suggestive of autoimmunity or immune dysregulation were identified in 14 patients (25.0%; **Supplementary Table 2**). These immune-related conditions were observed across all molecular subgroups, recapitulating overall distribution of CFCS-associated mutations. Atopic dermatitis was the most common manifestation, affecting 9 patients (17.0%). Atopic dermatitis was generally mild and managed with topical corticosteroids as needed. However, no additional data, particularly regarding allergic screening, were available. Other reported manifestations included psoriasis and celiac disease, each identified in two individuals (3.6%; one with a *BRAF* and one with a *MAP2K1* mutations, respectively). Less frequent presentations included seronegative autoimmune hepatitis, eosinophilic esophagitis, and thyroid disease, each occurring in one patient each (1.8%). Seronegative Autoimmune hepatitis was confirmed by liver biopsy in one patient with a *BRAF* mutation. Eosinophilic esophagitis and thyroid disease occurred in two individuals, both carrying *BRAF* mutations.

Notably, autoimmune cytopenia was not documented in our cohort. Thymectomy (either partial or total) was not reported.

### 3.5 Lymphoproliferation and malignancies

No cases of malignant neoplasms or polyclonal benign lymphoproliferation were identified in the whole cohort.

### 3.6 Immunological evaluation

The immune profile of the cohort was investigated by collecting CBC with differential, lymphocyte subsets and immunoglobulin levels (**Table 3**). Among the CBC results, hemoglobin levels were predominantly normal (86.7%), with a small subset showing low levels (9.4%). White blood cell counts were within normal range in most patients (76.9%), although leukocytosis was observed in 21.2%. Neutrophil counts were normal in nearly all cases (94.4%), while lymphopenia was present in 21.3% of individuals. Monocyte counts showed a trend toward monocytosis (32.4%), and platelet counts were mostly normal (90.4%) with occasional thrombocytosis (9.6%).

**Table 3:** Immune-hematological findings in the whole cohort.

	Patients, n	Low, n	%	Normal, n	%	High, n	%
Complete blood count with differential							
Hemoglobin	53	5	9.4	46	86.7	2	3.8
White blood cells	52	1	1.9	40	76.9	11	21.2
Neutrophils	36	1	2.8	34	94.4	1	2.8
Lymphocytes	47	10	21.3	34	72.3	3	6.4
Monocytes	34	0	0	23	67.6	11	32.4
Platelets	52	0	0	47	90.4	5	9.6
Lymphocyte subsets							
CD3+	43	8	18.6	35	81.4	0	0
CD4+	43	12	27.9	30	69.8	1	2.3
CD4+ naïve	11	0	0	11	100	0	0
CD8+	43	11	25.6	31	72.1	1	2.3
CD19+	44	5	11.4	37	84.1	2	4.6
CD19+ transitional	6	2	33.3	2	33.3	2	33.3
CD19+ naïve	11	6	54.6	2	18.2	3	27.3
CD19+ sm	12	4	33.3	8	66.7	0	0
CD3-CD16+CD56+	43	2	4.7	40	93.0	1	2.3
Immunoglobulins							
IgG	47	10	21.3	36	76.6	1	2.1
IgA	48	19	39.6	29	60.4	0	0
IgM	43	15	34.9	27	62.8	1	2.3

IgA, Immunoglobulin A; IgG, Immunoglobulin G; IgM, Immunoglobulin M; n = number of patients. Sm = switch memory B-cells.

Analysis of lymphocyte subsets revealed CD3+ T-cell lymphopenia in 18.6% of patients, with CD4+ lymphopenia in 27.9%, although naïve CD4+ T-cell counts were consistently normal in all tested patients. CD8+ T-cell deficiency was observed in 25.6% of cases. B-cell analysis showed reduced CD19+ cell numbers in 11.4% of patients, with a reduction in naïve B cells (54.6%) and switched memory B cells (33.3%). Natural killer (NK) cell counts were largely normal (93.0%). Twenty-six patients showed at least one decreased immunoglobulin isotype, specifically IgG (21.3%), IgA (39.6%), and IgM (34.9%). Decreased or absent response to vaccines was registered in 6 out of 16 patients (37.5%).

**Figure 2:**
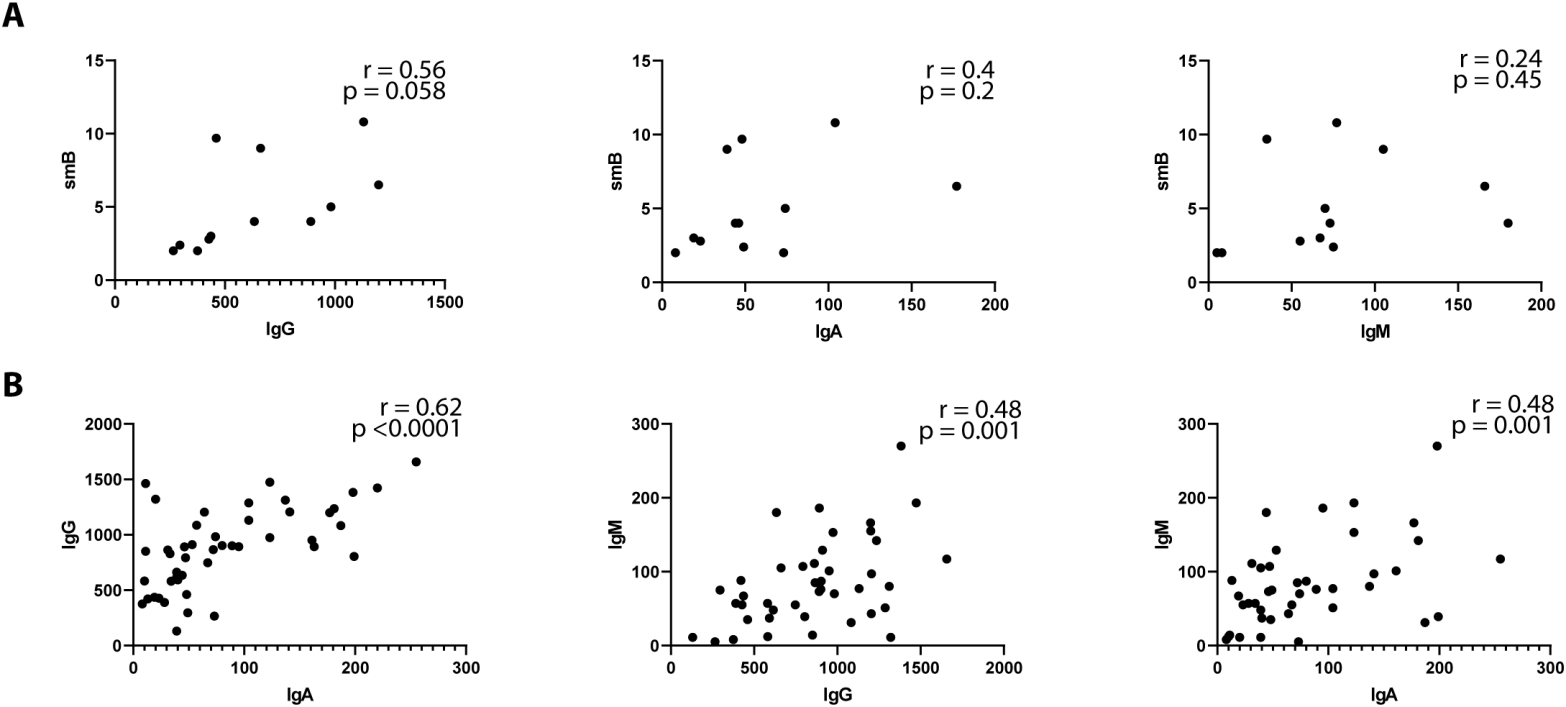
Immunoglobulins correlation profile in Cardiofaciocutaneous syndrome. Spearman’s correlation coefficient was used to compare between serum immunoglobulin isotypes and switched memory B (smB) cells **(A)** or immunoglobulin isotypes **(B)**. smB are depicted as percentages of total B cells. Immunoglobulin isotypes are depicted as per mg/ml.

No significant correlation was observed between the absolute number of B cells and the levels of IgG, IgA, or IgM (data not shown). However, a moderate correlation was noted between switched memory B (smB) cells and both IgG (r = 0.56, p = 0.058) and IgA (r = 0.40, p = 0.2), though these findings did not reach statistical significance. Among immunoglobulins, moderate correlations were identified between IgG and IgM (r = 0.48, p = 0.001), IgA and IgM (r = 0.48, p = 0.001), and IgG and IgA (r = 0.62, p < 0.0001), suggesting potential interdependencies among immunoglobulin levels, though the relationship with B-cell subsets warrants further investigation (**Figure 2**).

No statistically significant associations of hypogammaglobulinemia (either IgG, IgA or IgM), decreased IgG, IgM or IgA, or autoimmunity/immune dysregulation and infections were detected (data not shown). Similarly, no association between absent or partial vaccine responses and hypogammaglobulinemia or low smB was noticed (data not shown).

### 3.7 Genotype-phenotype correlation

Comparing *BRAF*- (n = 37) and *MAP2K1*-mutated (n = 14) patients revealed notable differences in several hematological and immunological parameters (**Table 4**). In the CBC, leukocytosis was observed more frequently in patients with *MAP2K1* (33.3%) compared to *BRAF* mutations (14.3%). Similarly, lymphocytosis was more common in the *MAP2K1* cohort (16.7%) compared to *BRAF* (3.1%), while lymphopenia was higher in *BRAF*-mutated patients (25.0%) than in *MAP2K1*-mutated patients (8.3%). Monocytosis was also more prevalent in *MAP2K1* patients (50.0%) compared to *BRAF* (21.7%).

**Table 4:** Comparison of immunological characteristics of *BRAF*- and *MAP2K1*-mutated patients.

	BRAF							MAP2K1						
Complete blood count														
	N	L	%	N	%	H	%		L	%	N	%	H	%
Hb	36	3	8.3	31	86.1	2	5.6	12	2	16.7	10	83.3	0	0.0
WBC	35	1	2.9	29	82.9	5	14.3	12	0	0.0	8	66.7	4	33.3
Neutrophils	25	1	4.0	24	96.0	0	0.0	8	0	0.0	7	87.5	1	12.5
Lymphocytes	32	8	25.0	23	71.9	1	3.1	12	1	8.3	9	75.0	2	16.7
Monocytes	23	0	0.0	18	78.3	5	21.7	8	0	0.0	4	50.0	4	50.0
Platelets	35	0	0.0	32	91.4	3	8.6	12	0	0.0	10	83.3	2	16.7
Lymphocyte subsets														
		L	%	N	N	H	H	M	L	%	N	N	H	H
CD3	29	6	20.7	22	75.9	0	0.0	11	1	9.1	10	90.9	0	0.0
CD4	29	10	34.5	18	62.1	1	3.5	11	1	9.1	10	90.9	0	0.0
CD4 naive	5	0	0.0	5	100	0	0.0	5	0	0.0	5	100	0	0.0
CD8	29	9	31.0	19	65.5	1	3.5	11	1	9.1	10	90.9	0	0.0
CD19	29	4	13.8	24	82.8	1	3.5	12	1	8.3	10	83.3	1	8.3
CD19 naive	5	3	60.0	2	40.0	0	0.0	5	2	40.0	0	0.0	3	60.0
CD19 smB	6	1	16.7	5	83.3	0	0.0	5	3	60.0	2	40.0	0	0.0
Natural Killer cells	29	2	6.9	26	89.7	1	3.5	11	0	0.0	11	100	0	0.0
Immunoglobulins														
		L	%	N	N	H	H		L		N	N	H	H
IgG	30	2	6.7	28	93.3	0	0.0	12	8	66.7	4	33.3	0	0.0
IgA	30	6	20,0	24	80.0	0	0.0	13	9	69.2	4	30.8	0	0.0
IgM	27	6	22.2	20	74.1	1	3.7	13	8	61.5	5	38.5	0	0.0

CBC, complete blood count; H, high; IgA, Immunoglobulin A; IgG, Immunoglobulin G; IgM, Immunoglobulin M; L, low; N, normal; smB, switched memory B cells; WBC, white blood cells.

Regarding lymphocyte subsets, CD4+ lymphopenia was observed in 34.5% of patients with *BRAF* mutations, whereas only 9.1% of *MAP2K1*-mutated individuals exhibited this finding. Similarly, CD8+ lymphopenia was more frequent in *BRAF*-mutated patients (31.0%) compared to *MAP2K1* (9.1%). A higher proportion of abnormalities in smB was found in *MAP2K1*-mutated individuals, with 60% showing decreased smB cells compared to 16.7% in BRAF-mutated patients.

Regarding immunoglobulin levels, IgG deficiency was observed in 6.7% of *BRAF*-mutated individuals compared to a much higher 66.7% in *MAP2K1* patients. Similarly, deficiencies in IgA (20.0% vs. 69.2%) and IgM (22.2% vs. 61.5%) were more prevalent in *MAP2K1*-mutated individuals.

Rate of recurrent/severe infections was similar between *BRAF*- (12/34) while 5 patients were *MAP2K1*-mutated patients (5/14; p>0.99).

The comparison of immune phenotypes between individuals harboring the Q257R *BRAF* mutation (n = 10) and those with the Y130C *MAP2K1* mutation (n = 6) highlighted notable differences (**Supplementary Table 3**). Lymphopenia was more common in Q257R patients (30.0%) compared to Y130C patients (20.0%). CD4+ lymphopenia was present in 33.3% of Q257R patients and 20.0% of Y130C patients, with the majority of patients in both groups maintaining normal CD8+ T-cell levels. Decreased B-cell counts were rare in Q257R patients (11.1%) but absent in Y130C patients. Decreased IgG levels were present in 11.1% of Q257R patients compared to 80% of Y130C patients, indicating a more severe defect in antibody production in the latter group. All the Y130C patients (100%) showed IgA deficiency, which was observed in 33.3% of Q257R patients. IgM deficiency was noted in 22.2% of Q257R patients compared to 83.3% of Y130C patients.

### 3.8 Treatment and overall survival

Given the widespread use of antiepileptic drugs (AEDs) in the CFC cohort, we investigated whether this treatment was associated with hypogammaglobulinemia as a potential side effect. Data regarding immunoglobulin levels was available for 18 out of the 24 patients receiving AEDs at the time of immunological evaluation. Among them, 50% (9/18) exhibited decreased levels of at least one immunoglobulin isotypes (IgG, IgA, or IgM). In the subgroup of patients not receiving AED treatment (32 individuals), immunoglobulin levels were assessed in 28 cases, with hypogammaglobulinemia detected in 17 cases. However, no statistically significant differences in immunoglobulin levels were observed between the AED-treated and non-treated groups (p = 0.55).

Immunodeficiency-related treatments were administered in 6 patients (10.7%). Immunoglobulin replacement therapy (IgRT) was initiated in three individuals, all of whom carried *MAP2K1* variants, had decreased IgG levels and a history of recurrent respiratory tract infections. Additionally, three patients received antibiotic prophylaxis, two due to recurrent respiratory infections (one of whom was also receiving IgRT) and due to a reduced absolute count of CD4+ T cells and naïve CD4+ T cells, for which trimethoprim-sulfamethoxazole was administered.

At the last follow-up visit, all the patients were alive.

## 4 Discussion

CFCS is a complex multisystem disorder with variable phenotypic expression. Building on the recent evidence reporting hypogammaglobulinemia in CFCS (8–10), here we describe a large international multicenter and molecularly defined cohort, providing the most comprehensive analysis of immune abnormalities in CFCS to date. We investigated immune dysregulation, susceptibility to infections, lymphocytes’ and immunoglobulins’ alterations, and treatments in affected individuals.

Our results demonstrate that immunological alterations are common in CFCS. The immunological phenotype is highly heterogeneous, ranging from asymptomatic patients to common variable immunodeficiency-like phenotypes. CFCS should be considered a syndromic immunodeficiency. Syndromic immunodeficiencies are conditions in which immunological defects may be present only in a subset of patients (16). In recent years, there has been growing interest not only in identifying novel syndromic immunodeficiencies (17–19) but also in reassessing known syndromic conditions to determine whether immunological defects have been previously overlooked (7, 20–23).

Lymphopenia, hypogammaglobulinemia, and infections emerged as recurrent features in CFCS. Although increased infection susceptibility, mostly involving the respiratory tract, was observed, opportunistic, viral or fungal severe infections were notably absent. The frequency of infections in our CFCS cohort appears lower than in other syndromic immunodeficiencies, such as in Kabuki syndrome (KS, 69%) (21), Jacobsen syndrome (JS, 50%) (22) or Rubinstein-Taybi Syndrome (RSTS, 72%) (7). While bronchiectasis, a hallmark of primary immunodeficiencies with hypogammaglobulinemia, was not reported in our cohort, it is important to note that pulmonary function testing and chest imaging were not routinely performed. Prospective studies will be needed to define the evolution and lung sequelae of recurrent infections in CFCS patients. Additionally, craniofacial anomalies, which are characteristic of CFCS, may contribute to an increased risk of recurrent upper and lower respiratory tract infections, further complicating the immunological phenotype. Despite the relatively mild impact of immune defects, 11% of our cohort required immunodeficiency-related treatments. Thus, immune evaluations should be considered in the clinical management of CFCS patients.

AEDs were commonly administered in our cohort. Although levetiracetam (24), carbamazepine (25), valproic acid (26) and phenytoin (27) have been implicated in causing hypogammaglobulinemia, we observed no significant differences in the prevalence of hypogammaglobulinemia between AED-treated and non-treated groups.

Despite the high prevalence of skin abnormalities among CFCS patients (21/36, 58.3%), seven of whom presented with nevi, no increased risk of melanoma was observed, consistent with previous reports (2). Unlike other RASopathies, which are classified as cancer-predisposing syndromes, our CFCS cohort did not exhibit malignant lymphoproliferative disorders, further distinguishing CFCS in terms of oncological risk (2).

A noteworthy finding in our study is the high incidence (25%) of immune-mediated manifestations, which have not been previously recognized as common complication of CFCS. Although immune-mediated manifestations could point toward primary immune-regulatory disorders (28), CFCS does not exhibit concomitant autoinflammation, lymphoproliferation, malignancy, and severe atopy. This suggests that, while CFCS features immune regulatory defects, it does not fit the classic definition of a primary immune regulatory disorder.

The diverse molecular landscape of CFCS contributes to the heterogeneity of its immunological phenotype. Oncogenic *BRAF* and *MAP2K1* mutations are functionally classified based on kinase activity, RAS-dependency and dimerization status (BRAF) (29, 30) or their dependence on phosphorylation by RAF for activation (MAP2K1) (31, 32). Germline *BRAF* mutations affecting the cysteine-rich domain (CRD; residues 234–280), such as the recurrent Q257R in our CFCS cohort, have been classified into CRD Classes A–C based on their ability to relieve autoinhibition and promote membrane binding. These mutations lead to enhanced RAS-dependent and -independent signaling and constitutive ERK activation (33). To date PVs or LPVs in the protein kinase domain of *BRAF* (F468-K601) (3) and p.Y130C/H/N variants of *MAP2K1* (exon 3) have both been associated with severe epileptic phenotype (34). These genotype-phenotype correlations underscore the importance of mutation-specific analyses in CFCS to refine clinical management strategies. Our findings highlight distinct immunological signatures associated with different causative mutations, particularly between *BRAF* and *MAP2K1* variants. *BRAF* mutations clustering in the CRD, particularly the Q257R variant, were predominantly associated with lymphopenia—especially T-cell lymphopenia—whereas *MAP2K1* mutations, particularly Y130C variant, were frequently linked to monocytosis, reduced naïve and switched-memory B cells, and hypogammaglobulinemia, suggesting differential effects on lymphocyte development and immune cell homeostasis driven by dysregulated MAPK signaling.

The mechanism by which a subset of CFCS patients shows immune alteration remains to be elucidated. The RAS/MAPK signaling pathway is fundamental for cellular homeostasis, differentiation, proliferation, and survival. To the best of our knowledge, the role of CFCS-associated proteins in the physiology of the immune system function has not been yet characterized. CFC-causing mutations dysregulate MAPK signaling resulting in increased ERK phosphorylation (35). Similar ERK hyperactivation is observed in other RASopathies, including CBL syndrome, where monocytes exhibit ERK hyperphosphorylation (36). CBL syndrome commonly shows monocytosis, increased transitional B cells and B-cell lymphocytosis (37). Although in our cohort monocytosis was a frequent finding, we were unable to systematically investigate transitional B cells, yet B cell lymphocytosis did not appear as a common feature. *In vitro* studies of *MAP2K1* mutations (K57M, G128V, Y130C) in neuroblastoma cell lines have demonstrated increased cell proliferation and autophagy (38). Given the tightly regulated metabolic demands of highly proliferative cells, including B cells and other hematopoietic cells, metabolic dysfunction is emerging as a key contributor to IEI (7, 19, 23). Notably, mitochondrial dysfunction, affecting oxidative phosphorylation and mitochondrial proteostasis, has been described in fibroblasts from patients with RASopathies, including CFCS (39, 40). These intriguing findings highlight the possibility that RAS/MAPK signaling dysregulation in CFCS may impact immune cell energy metabolism, contributing to the observed immunological abnormalities in CFCS’ hematopoietic cells.

Currently, no targeted therapies exist for CFCS. MEK inhibitors, including trametinib, have shown promising results in treating RASopathy-associated hypertrophic cardiomyopathy (41) and severe lymphatic anomalies (42–44). As MEK inhibitors are likely to be used in additional CFCS patients in the future, it would be valuable to conduct immunological assessments before and after treatment. Studying peripheral blood immune cells from CFCS patients undergoing MEK inhibition could provide critical insights into the role of RAS/MAPK signaling in hematopoietic cell function.

This study has several limitations. Approximately 35% of the patients were identified through literature review, leading to missing or incomplete data. Most published studies focus on pediatric populations, raising the possibility that immune system abnormalities (such as infections, immune dysregulation, and lymphoproliferation) may become more pronounced with age. Consequently, immune defects in CFCS could be underrecognized, and longitudinal studies are needed to fully assess their progression. Microbiological data, including pathogen identification and cultures during infectious episodes, were not systematically collected, limiting our ability to fully characterize the infectious burden and its etiology in CFCS patients.

Additionally, dual molecular diagnoses or multilocus genomic variations have been reported in up to 5% of patients undergoing whole exome sequencing (45) and in 10% of those screened for IEI (46). Since WES was not widely performed in our cohort, we cannot exclude the possibility that some patients carry additional pathogenic variants contributing to their immunological phenotype (47).

## 5 Conclusions

Until recently, the connection between immunological alterations and CFCS has been reported only in a few cases, and immunodeficiency was not listed within the main symptoms that individuals with CFCS may present. Our study establishes that CFCS is associated with variable but recurring immunological abnormalities, including lymphopenia, hypogammaglobulinemia, and increased infection susceptibility. Although immune defects in CFCS appear mild compared to other syndromic immunodeficiencies, a subset of patients requires immunodeficiency-related treatments. Our findings emphasize the need for routine immunological assessments in CFCS and highlight the potential role of RAS/MAPK signaling in immune system regulation. Future studies should focus on the metabolic and signaling pathways underlying CFCS-associated immune dysfunction, as well as the impact of MEK inhibitors on immune homeostasis.

## Data Availability

The datasets presented in this study can be found in online repositories. The names of the repository/repositories and accession number(s) can be found in the article/**Supplementary Material**.

## Supplementary material

Supplementary Table 1 Clinical, molecular and laboratory characteristics of the CFCS patients.

Supplementary Table 2 Immunological characteristics of *BRAF* Q257R vs *MAP2K1* Y130C mutated patients.
