# Supplementary material for: Cardiofaciocutaneous syndrome and immunodeficiency: data from an international multicenter cohort
Source: Front Immunol. 2025 Jul 7;16:1598896. doi: 10.3389/fimmu.2025.1598896 (PMC12277328; doi:10.3389/fimmu.2025.1598896)
Supplement: Supplementary file 1 [file DataSheet1.docx]

**Cardiofaciocutaneous Syndrome (CFCS) and Immunodeficiency: Data From an International Multicenter Cohort**

Benedetta Elena Di Majo^1,2^§, Chiara Leoni^3^§, Eleonora Cartisano^1,2^, Chiara Fossati^1^, Germana Viscogliosi^3^, Valentina Trevisan^3^, Lucia Pia Bruno^2^, Francesca Conti^4^, Mattia Moratti^4^, Emilia Monaco^3^, Donato Rigante^5,6^, Beatrice Rivalta^7^, Caterina Cancrini^7^, Aleksandra Szczawińska-Popłonyk^8^, Aleksander Jamsheer^9^, Monika Obara-Moszyńska^10^, Viktoria Zakharova^11^, Anna Shcherbina^12^, Julija Rodina^12^, Beyhan Tüysüz^13^, Saumya Shekhar Jamuar^14^, Jiin Ying Lim^14^, Jeannette Goh^14^, Anna Cereda^15^, Teresa Agovino^15^, Ilaria Contaldo^16^, Maria Luigia Gambardella^16^, Adriana Cristina Balduzzi^1,2^, Alessia Cherubino^3^, Giovanni Antonio Marrocco^3^, Silvia Bellesi^17^, Valentina Carusi^18^, Gabriele Rumi^19^, Andrea Biondi^20^, Giuseppe Zampino^3,6^, Francesco Saettini^1,20^*

§ These authors equally contributed to the manuscript

^1^ Fondazione IRCCS San Gerardo dei Tintori Hospital, Monza, Italy

^2^ Dipartimento Di Medicina e Chirurgia, Università Degli Studi Milano-Bicocca, Monza, Italy

^3^ Center for Rare Diseases and Birth Defects, Departmen of Woman and Child Health and Public Health, Fondazione Policlinico A. Gemelli, IRCCS, Rome, Italy

^4^ Pediatric Unit, IRCCS Azienda Ospedaliero-Universitaria di Bologna, Bologna, Italy

^5^ Department of Life Sciences and Public Health, Fondazione Policlinico Universitario A. Gemelli IRCCS, Rome, Italy

^6^ Università Cattolica Sacro Cuore, Rome, Italy

^7^ Pediatric Unit, Immunological and infectious diseases Unit, Primary immunodeficiency research Unit, Bambino Gesù Pediatric Hospital, Rome, Italy

^8^ Department of Pediatric Pneumonology, Allergy and Clinical Immunology, Institute of Pediatrics,

Poznań University of Medical Sciences, Poznań, Poland

^9^ Department of Medical Genetics, Poznań University of Medical Sciences, Poznań, Poland

^10^ Department of Biostatistics and Translational Medicine, Medical University of Lodz, Poland

^11^ Clinical Data Analysis Department, National Medical Research Center for Endocrinology, Moscow, Russian Federation

^12^ Department of Immunology, Dmitry Rogachev National Research Center of Pediatric Hematology, Oncology and Immunology, Moscow, Russian Federation

^13^ Department of Pediatric Genetics, Cerrahpasa Medical School, Istanbul University-Cerrahpasa, Istanbul, Turkey

^14^ Genetics Service, KK Women’s and Children's Hospital, Singapore, Singapore

^15^ Department of Pediatric, "Papa Giovanni XXIII" Hospital, Bergamo, Italy

^16^ Child Neurology and Psychiatric Unit, Department of Woman and Child Health and Public Health, Fondazione Policlinico Univaersitario A. Gemelli, IRCCS, Rome, Italy

^17^Dipartimento di Scienze di Laboratorio ed Ematologiche, Fondazione Policlinico Gemelli, IRCCS, Rome, Italy

^18^ UOSD Allergologia ed Immunologia Clinica, Dipartimento Scienze Mediche e Chirurgiche, Fondazione Policlinico Universitario A. Gemelli, IRCCS, Rome, Italy

^19^ Unità Operativa Semplice Malattie Infiammatorie Croniche Intestinali, IBD Unit, CEMAD, Fondazione Policlinico Universitario A. Gemelli, IRCCS, Rome, Italy

^20^ Centro Tettamanti, Fondazione IRCCS San Gerardo dei Tintori, Monza, Italy

*** Correspondence:**Francesco Saettini

f.saettini@gmail.com

**Keywords: primary immunodeficiency, inborn errors of immunity, cardiofaciocutaneous syndrome, RASopathy, hypogammaglobulinemia, BRAF, MAP2K1, syndromic immunodeficiency.**

**Supplementary Figure 1. PRISMA flowchart of publication retrieval, inclusion and exclusion of papers.**

**
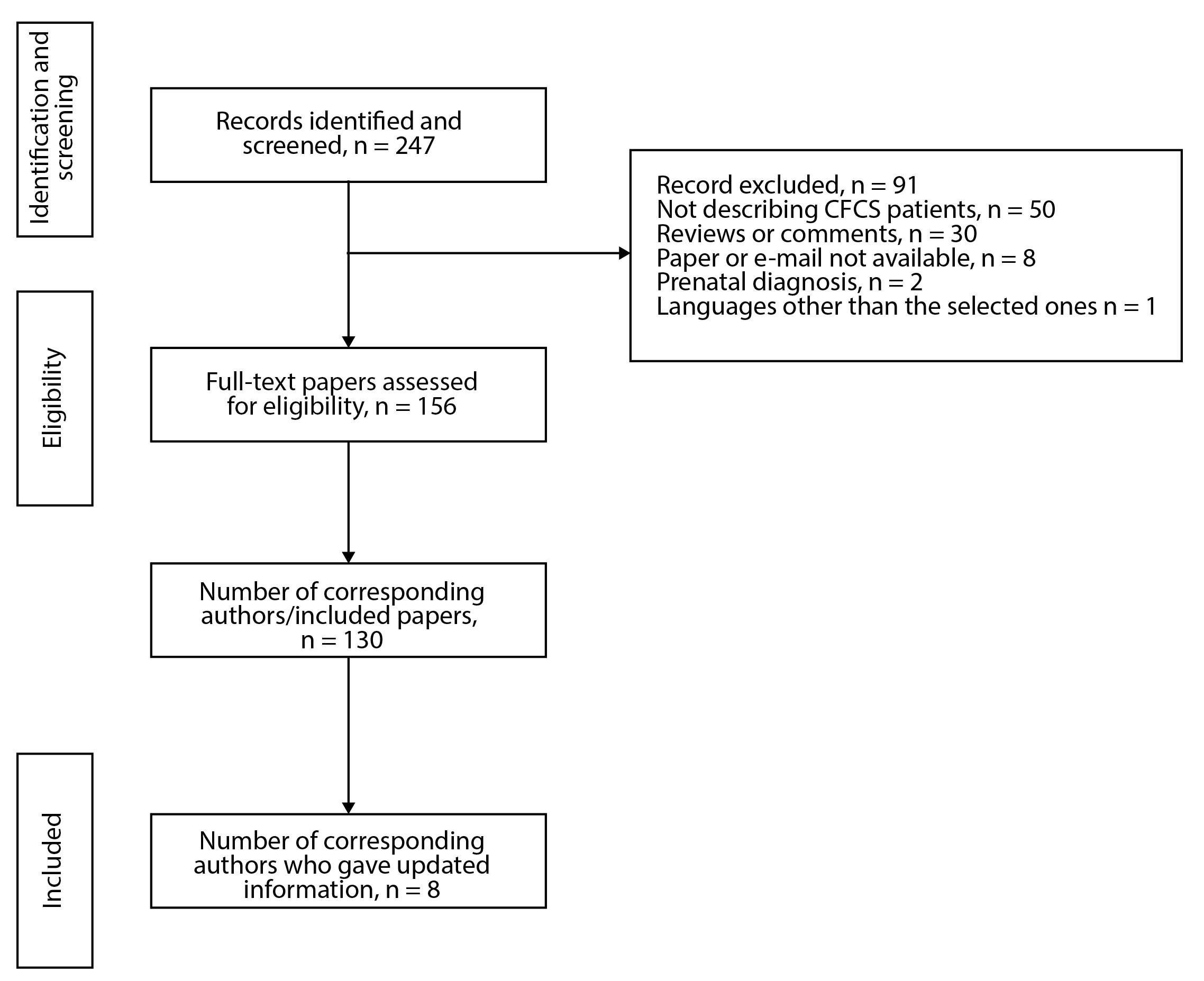
**

**Supplementary Table 2. Autoimmune characteristics in patients with cardiofaciocutaneous syndrome.**

|  | **Number of patients (total)** | **%** |
| --- | --- | --- |
| **Autoimmune manifestations**  *BRAF*  *MAP2K1*  *MAP2K2*  *KRAS*  *YWHAZ* | **14 (56)**  8 (14)  3 (14)  1 (14)  1 (14)  1 (14) | **25.0**  57.1  21.4  7.1  7.1  7.1 |
| **Atopic dermatitis**  *BRAF*  *MAP2K1*  *MAP2K2*  *KRAS*  *YWHAZ* | **9 (56)**  5 (9)  2 (9)  1 (9)  0 (9)  1 (9) | **16.1**  55.6  22.2  11.1  0.0  11.1 |
| **Psoriasis**  *BRAF*  *KRAS* | **2 (56)**  1 (2)  1 (2) | **3.6**  50.0  50.0 |
| **Coeliac Disease**  *BRAF*  *MAP2K1* | **2 (56)**  1 (2)  1 (2) | **35.7**  50.0  50.0 |
| **Autoimmune hepatitis**  *KRAS* | **1 (56)**  1 (1) | **1.8**  100 |
| **Eosinophilc Esophagitis**  *BRAF* | **1 (56)**  1 (1) | **1.8**  100 |
| **Thyroid disease**  *BRAF* | **1 (56)**  1 (1) | **1.8**  100 |

**SUPPLEMENTARY MATERIAL**

**Supplementary** **Table 3. Immunological characteristics of *BRAF* Q257R vs *MAP2K1* Y130C mutated patients.**

|  | ***BRAF* Q257R** | | | | | | | ***MAP2K1* Y130C** | | | | | | |
| --- | --- | --- | --- | --- | --- | --- | --- | --- | --- | --- | --- | --- | --- | --- |
|  | **N** | **L** | **L** | **N** | **N** | **H** | **H** | **N** | **L** |  | **N** | **N** | **H** | **H** |
| **CBC** | 10 | 0 | 0.0 | 10 | 100 | 0 | 0.0 | 4 | 1 | 25.0 | 3 | 75.0 | 0 | 0.0 |
| Hb | 10 | 0 | 0.0 | 9 | 90.0 | 1 | 10.0 | 4 | 0 | 0.0 | 4 | 100 | 0 | 0.0 |
| WBC | 6 | 0 | 0.0 | 6 | 100 | 0 | 0.0 | 3 | 0 | 0.0 | 3 | 100 | 0 | 0.0 |
| Neutrophils | 10 | 3 | 30.0 | 7 | 70.0 | 0 | 0.0 | 5 | 1 | 20.0 | 4 | 80.0 | 0 | 0.0 |
| Lymphocytes | 6 | 0 | 0.0 | 5 | 83.3 | 1 | 16.7 | 3 | 0 | 0.0 | 3 | 100 | 0 | 0.0 |
| Monocytes | 10 | 0 | 0.0 | 9 | 90.0 | 1 | 10.0 | 4 | 0 | 0.0 | 3 | 75.0 | 1 | 33.3 |
| **Lymphocyte subsets** |  | L | L | N | N | H | H |  | L | L | N | N | H | H |
| CD3 | 9 | 1 | 11.1 | 8 | 88.9 | 0 | 0.0 | 5 | 1 | 20.0 | 4 | 80.0 | 0 | 0.0 |
| CD4 | 9 | 3 | 33.3 | 6 | 66.7 | 0 | 0.0 | 5 | 1 | 20.0 | 4 | 80.0 | 0 | 0.0 |
| CD4 NAIVE | 2 | 0 | 0.0 | 2 | 100 | 0 | 0.0 | 2 | 0 | 0.0 | 2 | 100 | 0 | 0.0 |
| CD8 | 9 | 1 | 11.1 | 8 | 88.9 | 0 | 0.0 | 5 | 1 | 20.0 | 4 | 80.0 | 0 | 0.0 |
| CD19 | 9 | 1 | 11,1 | 8 | 88.9 | 0 | 0.0 | 6 | 0 | 0.0 | 6 | 100 | 0 | 0.0 |
| CD19 NAIVE | 2 | 2 | 100 | 0 | 0.0 | 0 | 0.0 | 2 | 1 | 50.0 | 0 | 0.0 | 1 | 50.0 |
| CD19 smB | 2 | 0 | 0.0 | 2 | 100 | 0 | 0.0 | 2 | 1 | 50.0 | 1 | 50.0 | 0 | 0.0 |
| Natural Killer cells | 9 | 0 | 0.0 | 9 | 100 | 0 | 0.0 | 5 | 0 | 0.0 | 5 | 100 | 0 | 0.0 |
|  |  |  |  |  |  |  |  |  |  |  |  |  |  |  |
| **Ig** |  | **L** | **L** | **N** | **N** | **H** | **H** |  | **L** | **L** | **N** | **N** | **H** | **H** |
| IgG | 9 | 1 | 11.1 | 8 | 88.9 | 0 | 0.0 | 5 | 4 | 80.0 | 1 | 20.0 | 0 | 0.0 |
| IgA | 9 | 3 | 33.3 | 6 | 66.7 | 0 | 0.0 | 6 | 6 | 100 | 0 | 0.0 | 0 | 0.0 |
| IgM | 9 | 2 | 22.2 | 7 | 77.8 | 0 | 0.0 | 6 | 5 | 83.3 | 1 | 16.7 | 0 | 0.0 |

CBC= complete blood count; H= high; IgG= Immunoglobulin G; IgA= Immunoglobulin A; IgM= Immunoglobulin M; N= normal; L= low; WBC= white blood cells
